# Improving emergency medicine resident pediatric lumbar puncture procedural performance through a brief just-in-time video intervention

**DOI:** 10.1186/s12909-024-05654-1

**Published:** 2024-06-20

**Authors:** Sarayna S. McGuire, Alexander S. Finch, Jenna M. Thomas, Octavio Lazaro, Sara A. Hevesi, Aidan F. Mullan, Jim L. Homme

**Affiliations:** 1 Department of Emergency Medicine, Mayo Clinic, 200 First St SW, Rochester, MN 55905 USA; 2 Department of Emergency Medicine, Washington University School of Medicine, St Louis, MO USA; 3 Department of Quantitative Health Sciences, Mayo Clinic, Rochester, MN USA

**Keywords:** Lumbar puncture, Procedural efficiency, Resident education, Emergency medicine, Pediatric lumbar puncture, Pediatric procedure, Medical education, Just-in-time education

## Abstract

**Background:**

Emergency medicine (EM) trainee comfort level with lumbar puncture (LP) has decreased over time due to changing practice guidelines, particularly amongst pediatric patients. We implemented a “just in time” (JIT) brief educational video based on a previously published LP Performance Scoring Checklist to improve trainee efficiency and competence in LP performance.

**Methods:**

Our pilot quasi-experimental study took place January-June 2022 within a large, academic Midwestern emergency department (ED) with an established 3-year EM residency program. All 9 interns performed a timed diagnostic LP on an infant LP model in January, scored according to the LP Performance Scoring Checklist. In June, interns repeated the timed LP procedure directly after watching a brief educational video based on major checklist steps. The study was deemed exempt by the Institutional Review Board.

**Results:**

All interns completed both assessments. At baseline, interns had logged performance of median 2 (IQR 0–5) LPs and spent 12.9 (10.3–14.4) minutes performing the procedure. Post-intervention, interns had logged an additional median 2 (0–5) LPs and completed the procedure faster with an average time of 10.3 (9.7–11.3) minutes (*p* = 0.004). A median of 5 (4–7) major steps were missed at baseline, compared to 1 (1–2) at time of post-intervention assessment (*p* = 0.015).

**Conclusion:**

Development of a brief educational video improved efficiency and competency amongst our intern class in performing an infant LP when viewed Just-In-Time. Similar efforts may improve education and performance of other rare (or decreasing in frequency) procedures within EM training.

## Background

The Accreditation Council for Graduate Medical Education (ACGME) requires emergency medicine (EM) residencies to certify competence of trainees in procedures viewed as essential to the independent practice of EM [1]. Lumbar puncture (LP) is one of these procedures as it remains a mainstay of the diagnostic evaluation for patients with suspected central nervous system disorders due to infection, autoimmunity, and hemorrhage, and provides diagnostic and therapeutic benefit in cases such as idiopathic intracranial hypertension [2].

### Importance

Despite its importance, trainee comfort level with the LP procedure has decreased over time due to changing practice guidelines, particularly amongst pediatric patients [3]. Across children’s hospitals nationwide, performance of LPs has decreased by 37% over the past decade, likely due to widespread vaccination and implementation of risk stratification algorithms that have changed the way febrile infants are managed in the emergency department (ED) [4]. Additionally, the role of lumbar puncture in the investigation of sudden onset headache has declined with changing imaging technology and utilization of contrast angiography [5]. With decreasing clinical experience during training, average attending experience with the procedure is also expected to decrease, making effective standardized training interventions ever more important in competency-based medical education.

### Goals of this investigation

We sought to improve competency (mastery of specific knowledge and skills) [6] of this increasingly rare procedure in our residency through a brief educational video, focusing initial efforts on our post-graduate year (PGY)-1 intern class.

## Methods

### Study design and setting

This pilot quasi-experimental study took place January—June 2022 within a large, academic Midwestern ED. The ED has an established 3-year EM residency with 9 residents per year and features both adult and pediatric care areas. Within the ED, 32 LPs were performed on pediatric (< 18 years of age) patients between 7/1/2021 and 6/30/2022, 25 (78.1%) on patients < 1 year of age. EM residents performed 17 (53.1%) of the total pediatric LPs, including 13 (52.0%) of the infant LPs.

### Selection of participants

In January, all 9 EM interns were invited to participate in a “procedural project,” with the type of procedure blinded to participants. Participants were compensated with a $10 hospital cafeteria voucher, with an additional $5 bonus voucher offered if the entire class participated. The same compensation was offered at the time of post-intervention assessment in June. Informed consent of participants was obtained at time of assessment scheduling and re-obtained verbally at time of assessment performance. The study was reviewed by the Mayo Clinic Institutional Review Board and deemed exempt.

### Baseline measurement

**Fig. 1:**
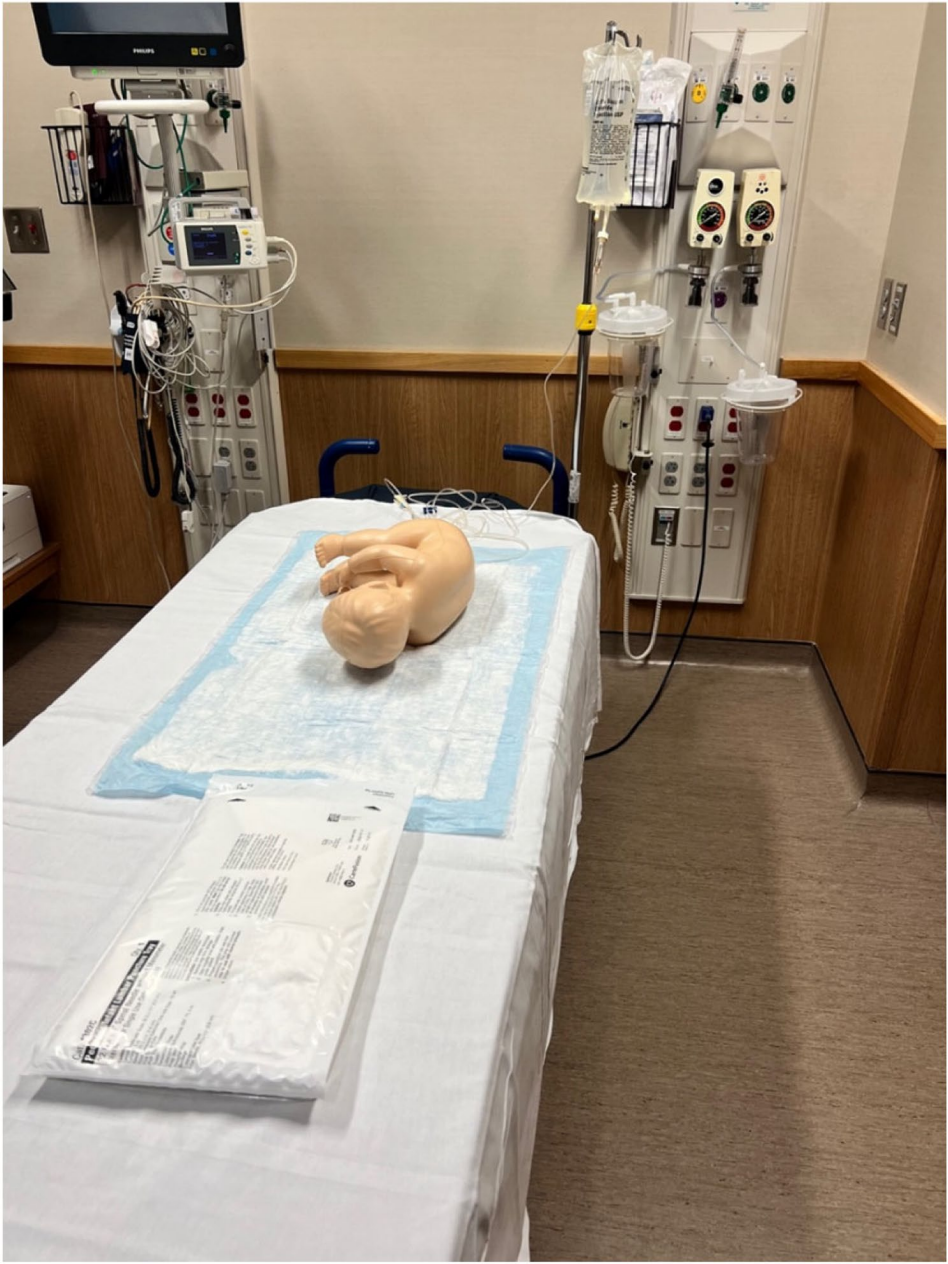
Procedural Performance Assessment Setup with Pediatric LP Simulator II Model [7] and CareFusion Pediatric/Infant LP Tray

At their individual baseline performance assessment, participants were provided the department’s standard CareFusion Pediatric/Infant LP Kit and asked to perform a timed diagnostic LP (including obtaining an opening pressure) on an infant LP model (Pediatric LP Simulator II Model) [7]. Cerebrospinal fluid (CSF) was simulated by a hanging liter bag of saline connected to a rubber tube concealed within the model, allowing for flow with successful dura puncture (Fig. 1). This simulated CSF flow, along with a tactile “pop” sensation with needle entry into the tube, allowed for recognition of penetration of the dura. Non-sheer tape was replaced over the spine before each trainee to obscure prior puncture marks and ensure the tactile pop sensation was maintained with each attempt.

Performance was scored according to a previously published LP Performance Scoring Checklist as described in Lammers et al. in which the procedure and scoring system were validated by a convenience sample of emergency physicians experienced with the LP procedure [3] Participants were asked to obtain opening pressure consistent with the checklist and to test competence as this is a key diagnostic part of LPs in certain circumstances. A PGY-3 EM chief resident (S.M.) and EM attending physician (A.F.) observed and scored the assessments to assess competence (critical actions correctly performed) utilizing the checklist’s 26 major steps (Table 1), defined by Lammers et al. as “critical steps that could cause a complication or procedural failure if omitted, performed incorrectly, or performed out of sequence.” [3] Lammers’ 44 minor steps were omitted as some were optional variances in technique [3]. Participants were encouraged to verbalize their actions and were prompted, when necessary, to clarify details associated with a step (e.g. which bony landmark they were palpating and to which interspace level it corresponded). Both S.M. and A.F. independently scored each participant during the session and any disagreements in scoring were resolved through discussion and consensus. Timing began after instructions were given and ended after a bandage was placed and caps screwed onto all collection tubes. Participants received no feedback during or after the procedure and did not view the checklist being utilized.

**Table 1:** Completion of Major Steps (from previously published Performance Scoring Checklist to assess Competence in Lumbar Punctures) [3] at Baseline Procedural Performance Assessment compared to Post-Intervention Procedural Performance Assessment (*N* = 9)^†^

			Time to completion (minutes)		
Validation item/ major step	Description	Step performed	Baseline 12.9 (10.3, 14.4)	Post-intervention 10.3 (9.7, 11.3)	*p*-value 0.004
1.1	Place the patient in a lateral decubitus position or upright position.	No Yes	0 (0.0%) 9 (100%)	0 (0.0%) 9 (100%)	---
1.2	Check the spine for maximum flexion.	No Yes	8 (89%) 1 (11%)	6 (67%) 3 (33%)	0.35
2.1	Identify the L4-L5 interspace at the point intersecting the iliac crest line with the body midline.	No Yes	3 (33%) 6 (67%)	1 (11%) 8 (89%)	0.35
3.1	Put on sterile gloves without contamination.	No Yes	1 (11%) 8 (89%)	0 (0%) 9 (100%)	0.99
4.1	Place sponge stick into Betadine.	No Yes	0 (0.0%) 9 (100%)	0 (0.0%) 9 (100%)	---
4.2	Wipe the skin in a circular motion from the target area to about a 10 cm radius.	No Yes	0 (0.0%) 9 (100%)	0 (0.0%) 9 (100%)	---
5.1	Insert the needle into the subcutaneous tissue.	No Yes	2 (22%) 7 (78%)	0 (0%) 9 (100%)	0.35
5.2	Inject 1–3 cc of anesthetic solution.	No Yes	0 (0.0%) 9 (100%)	0 (0.0%) 9 (100%)	---
6.1	Place the needle in the center of the interspace.	No Yes	0 (0.0%) 9 (100%)	0 (0.0%) 9 (100%)	---
6.2	Angle the needle toward the umbilicus.	No Yes	2 (22%) 7 (78%)	0 (0%) 9 (100%)	0.35
7.1	Advance the needle into the skin slowly and smoothly.	No Yes	5 (56%) 4 (44%)	0 (0%) 9 (100%)	0.037
7.2	Once the needle passes through the subcutaneous tissue, turn the bevel of the needle laterally.	No Yes	0 (0%) 9 (100%)	3 (33%) 6 (67%)	0.15
7.3	Advance the needle.	No Yes	0 (0.0%) 9 (100%)	0 (0.0%) 9 (100%)	---
7.4	Remove the stylet and check for fluid.	No Yes	3 (33%) 6 (67%)	0 (0%) 9 (100%)	0.15
7.5	Reinsert the stylet.	No Yes N/A	1 (11%) 8 (89%) 0 (0%)	0 (0%) 7 (78%) 2 (22%)	0.99
7.6	Advance the needle further until a pop is felt, an obstruction prevents further movement or the patient reports parasthesias or radicular pain.	No Yes N/A	3 (33%) 6 (67%) 0 (0%)	1 (11%) 5 (56%) 3 (33%)	0.60
7.7	Remove the stylet and check for fluid.	No Yes N/A	1 (11%) 8 (89%) 0 (0%)	0 (0%) 6 (67%) 3 (33%)	0.99
7.8	If there is an obstruction and no fluid or if there are parasthesias and no fluid or if there is bright red blood, withdraw the needle and repeat the above 2 steps, reposition the needle or use a different interspace.	No Yes N/A	3 (33%) 3 (33%) 3 (33%)	0 (0%) 2 (22%) 7 (78%)	0.46
8.1	Attach the manometer/stopcock to the needle hub.	No Yes	2 (22%) 7 (78%)	1 (11%) 8 (89%)	0.77
8.2	Turn the stopcock valve until the dial is parallel with the manometer.	No Yes	5 (56%) 4 (44%)	1 (11%) 8 (89%)	0.072
8.3	Allow the fluid to fill the manometer until the meniscus stops rising.	No Yes	0 (0.0%) 9 (100%)	0 (0.0%) 9 (100%)	---
8.4	Measure the CSF opening pressure correctly.	No Yes	0 (0.0%) 9 (100%)	0 (0.0%) 9 (100%)	---
9.1	Place the first tube under the stopcock.	No Yes	5 (56%) 4 (44%)	1 (11%) 8 (89%)	0.072
9.2	Collect 1 cc of CSF.	No Yes	3 (33%) 6 (67%)	1 (11%) 8 (89%)	0.35
9.3	Screw the cap on the first tube with one hand, and place the tube upright in the slot on the tray.	No Yes	3 (33%) 6 (67%)	0 (0%) 9 (100%)	0.15
10.1	Withdraw the needle.	No Yes	2 (22%) 7 (78%)	1 (11%) 8 (89%)	0.99

*Source*: Used with permission from John Wiley and Sons

^†^Items marked as “N/A” (Not Applicable) indicate the participant successfully punctured the dura with CSF flow observed by step 7.4, thereby not requiring subsequent steps 7.5–7.8. P-values comparing baseline and post-intervention assessments do not include “N/A” responses

### Intervention

S.M. and O.L. designed and filmed a brief (4.25 min) instructional LP procedure video [8] that demonstrated the checklist’s major steps (Table 1). The video was filmed over a two-hour session in the ED with a Sony Alpha (Sony Corporation, Tokyo, Japan) camera and edited over approximately six hours with Wondershare Filmora (Wondershare Technology, Shenzhen, China) editing software. The video was distributed via email in March to all trainees rotating through the ED and uploaded onto the department’s Intranet Education webpage.

### Post-intervention measurement

At the time of post-intervention assessment, participants were asked whether they had reviewed or utilized the video. Regardless of response, all participants were shown the video before proceeding with the same procedural assessment on the same infant LP model as performed at baseline, described above. This was done to ensure that the video was used as a JIT resource, as intended, rather than viewed at a time and location remote from procedural performance.

The number of LPs logged by participants, as required by the residency, were pulled from the program’s graduate medical education (GME) administrative management software [9] in order to ascertain LP procedural experience at time of each assessment. Logged procedures could include those performed in the clinical and simulation settings.

### Data analysis

Intern performance on the LP Performance Scoring Checklist [3] was compared between baseline and post-intervention assessments using paired Fisher’s exact tests (completion of each major step) and Wilcoxon signed-rank tests (time to completion, total steps missed).

## Results

All 9 interns completed both the baseline and post-intervention performance assessments. At the time of baseline assessment, interns had logged a median 4 (IQR 0–10) LPs, with median 2 (IQR 0–5) logged as “performed” (as opposed to observed or assisted). Median baseline time to completion of the procedure was 12.9 (10.3–14.4) minutes (Table 1). Post-intervention, interns had logged an additional median of 4 (0–11) LPs, including median 2 (IQR 0–5) logged as “performed” and completed the procedure faster with a median time of 10.3 (9.7–11.3) minutes (*p* = 0.004). Three (33.3%) of the interns reported previously reviewing the video on their own. A median of 5 (4–7) major steps were missed by each intern at baseline, compared to median 1 (1–2) major step missed at time of post-intervention assessment (*p* = 0.015).

Interns demonstrated either continued (*n* = 8) or improved competency (*n* = 17) of all major steps post-intervention, with the exception of turning the bevel of the needle laterally (Step 7.2). Among the major steps that were completed more frequently post-intervention, the only one reaching statistical significance was 7.1, advancement of the needle into the skin slowly and smoothly (*p* = 0.037).

## Discussion

Innovative approaches have historically been employed to provide medical trainees exposure to infrequent, invasive, and time-sensitive procedures which they may rarely, if ever, encounter [10–13]. As the frequency of LPs performed in the clinical environment decreases, so too do trainees’ encounters with them. Not only does the ACGME require residents to cite performance of a certain number of these procedures in order to graduate [1], GME in the United States has moved towards a competence-based approach [6], requiring educators collect and demonstrate measurable evidence that trainees achieve specific, predetermined outcomes [14, 15]. Hidden amongst trainee data is the implication that as experience during training with a clinical procedure decreases, so too does the experience of the next generation of attending physicians who are responsible for supervising trainees. With increasing focus on managing (and measuring) cognitive load in medical education for both trainer and trainee, educational interventions which target optimization of cognitive load may benefit clinical practice in both immediate and long-term settings [16, 17]. By utilizing a previously published checklist in a brief JIT video, we ensure that agreed-upon elements of procedural competence are reliably demonstrated to the trainee independent of the supervising physician’s time, attention, or skill in teaching, all of which are particularly variable in the clinical environment where other departmental and patient care priorities must be balanced. Future research is needed to determine whether supervising physicians do indeed perceive benefits of such JIT resources in their ability to precept and teach rare procedures on shift.

Our study demonstrates the benefit of utilizing a brief procedural video on both resident efficiency (time spent) and competence (critical actions correctly performed) in performing a LP. Given 66.7% of our cohort acknowledged watching the video for the first time at their post-assessment, our findings suggest a Just-In-Time (JIT) learning benefit amongst our small cohort. Given prior more rigorous JIT LP studies have demonstrated equivocal success [18, 19], our results may best demonstrate the ease in creating and implementing a JIT teaching tool for increasingly rare procedures such as a LP and that similar efforts may improve education and performance of other rare procedures within EM training. Our tracking of procedural completion time was for the purpose of determining comfort and familiarity with the procedure amongst our trainees with the belief that increased competency leads to a procedure performed smoothly and uninterrupted. This reflects prior literature on knowledge encapsulation, which has demonstrated encapsulating concepts in recall and increased level of expertise lead to efficiency in certain diagnostic targets [20–22]. Consistent with this, we did see improvement in both efficiency and competence in our cohort. We isolated the intern class as our intervention cohort given their expected novice baseline experience and competency with the procedure, as well as the need to improve upon both during their training. It is possible that the intervention had the same beneficial or a less beneficial impact amongst more senior EM residents or trainees from other specialties.

We used a six-month time period between assessments to balance adequate time for simulation skill decay against clinical encounters allowing for LP performance. Participants documented performing a median of 2 LPs (and overall completing a median of 4 LPs) during the six months between baseline and post-intervention assessments, which was similar to the six months prior to baseline assessment (median 4 LPs, performed: *p* = 0.81; completed: *p* > 0.99). This suggests that exposure to lumbar punctures external to our study likely had minimal influence on the improvement we observed in procedural efficiency and competency after our intervention. We do acknowledge the possibility that increased exposure to other procedures that require an organized stepwise approach that also includes manual dexterity could have some positive impact on lumbar puncture task performance.

Our study demonstrated benefit to trainee efficiency and competency after watching the video. However, only 1/3 of participants acknowledged having watched the video independently, and it is unclear to what degree attending physicians supervising these trainees utilized the video as a JIT tool as intended. Utilizing a brief video to provide JIT teaching and/or review may have the benefit of cognitively offloading the supervising physician during the clinical shift, decreasing the required time away from other clinical responsibilities while being certain that all standardized evaluation (and by extension, competency) criteria have been demonstrated to the trainee by the video. Moreover, this allows a brief “refresher” of the procedure for the supervising physician if desired, particularly if they have not recently performed the procedure themselves. More effort is warranted to demonstrate the effectiveness and ease of use of similar JIT training videos to all stakeholders (trainer and trainee alike) in the future in order to encourage greater uptake.

### Limitations

This study has several important limitations. Most salient is the small number of learners in our sample. We viewed this as a pilot to test whether a JIT video based on a previously published checklist for procedural evaluation was effective in achieving increased procedural efficiency and/or competence, and feasible to design and distribute within a cohort of junior learners. Given our desire to improve trainee performance with the LP procedure, overall, we chose a previously published generalized LP checklist [3] to use to create our JIT video. Lammers et al. describe utilization of a model consisting of a torso with palpable landmarks and simulated CSF fluid, however they do not specify whether the model was intended to be viewed by participants as an adult or child [3]. We maintained use of this checklist when assessing performance of our study participants. We acknowledge that this previously published generalized LP checklist may not maintain its validation in assessing performance of an LP on a simulated infant patient, although an infant-specific LP checklist by Auerbach et al. does include similar critical steps [23]. Additionally, we did not independently verify trainee procedure logs, which could allow for error in entry by the performing resident. Administrative habits were thought likely to remain stable between the time of both assessments. We presented overall data of procedure logs, including number of LPs logged as having been directly performed and those logged as having been completed overall. We are unable to verify how many of these ‘completed’ LPs were actually simulated, observed, or even supervised with performance feedback, nor are we able to verify how many were performed on the particular task trainer used in this study. Additionally, although we did not tell participants beforehand what procedure they would be asked to perform at time of their second (post-intervention) assessment, it is possible that they correctly assumed it would again be a LP and reviewed other resources to prepare. To mitigate this, we emphasized that performance would have no bearing on a participant’s residency evaluations. Although the simulator model used was high-quality, the static nature of our model does not fully replicate performing an LP procedure on a real patient. However, given the demonstrated decrease in availability of these procedures clinically, we believe our high-fidelity simulated model serves as a reasonable available alternative. Finally, this study was performed within one academic EM residency program in the Midwest and may not be generalizable broadly to other training programs or institutions.

## Conclusion

Development of a brief educational video improved efficiency and competency amongst our intern class in performing an infant LP when viewed Just-In-Time. Similar efforts may improve education and performance of other rare (or decreasing in frequency) procedures within EM training.

## Data Availability

The datasets used and analyzed in the study are available from the corresponding author on reasonable request.

## Declarations

### Meetings

An abstract with limited data from this study was presented at the CORD 2022 meeting in San Diego, CA.
